# How drinking motives mediate associations between sexual orientation and indicators of alcohol use – a study among young Swiss men

**DOI:** 10.3389/fpsyg.2024.1416062

**Published:** 2025-01-20

**Authors:** Matthias Wicki, Simon Marmet, Joseph Studer, Kim Bloomfield, Gerhard Gmel

**Affiliations:** ^1^Addiction Medicine, Lausanne University Hospital and University of Lausanne, Lausanne, Switzerland; ^2^Institute for Research, Development and Evaluation, Bern University of Teacher Education, Bern, Switzerland; ^3^Department of Psychiatry, North-West Vaud Adult Psychiatry Service, Lausanne University Hospital and University of Lausanne, Lausanne, Switzerland; ^4^Unit for Health Promotion Research, Department of Public Health, University of Southern Denmark, Copenhagen, Denmark; ^5^Centre for Alcohol and Drug Research, Aarhus University, Aarhus, Denmark; ^6^Institute of Biometry and Clinical Epidemiology, Charité - Universitätsmedizin Berlin, Berlin, Germany; ^7^Addiction Switzerland, Lausanne, Switzerland; ^8^Centre for Addiction and Mental Health, Toronto, ON, Canada; ^9^Alcohol and Health Research Unit, University of the West of England, Bristol, United Kingdom

**Keywords:** cohort study on substance use risk factors (C-SURF), alcohol use, sexual orientation, young adults, drinking motives

## Abstract

**Background:**

Individuals with a minority sexual orientation have consistently been found to face a greater risk of mental health problems and problematic substance use than heterosexual individuals. The present study examined whether differences in alcohol use or alcohol use disorder (AUD) symptoms across the spectrum of sexual orientations could be explained by drinking motives (i.e., enhancement, social, coping and conformity motives).

**Method:**

A non-self-selective sample of non-abstinent, young Swiss men (N = 5,139; mean age = 25.4, SD = 1.25) completed a self-reporting questionnaire on sexual orientation (on a five-point attraction scale: heterosexual, mostly-heterosexual, bisexual, mostly-homosexual, homosexual), drinking motives, alcohol use indicators (e.g., heavy episodic drinking, Alcohol Use Disorders Identification Test-Consumption [AUDIT-C]), and AUD symptoms. Structural equation modeling was used to test whether drinking motives mediated the associations between dummy-coded sexual orientation (with heterosexual men as the reference) and alcohol use indicators or AUD symptoms.

**Results:**

Mostly-heterosexual men exhibited higher scores on alcohol use indicators than heterosexual men, with almost full mediation through their drinking motives, specifically higher enhancement motives. They also reported more AUD symptoms, partially mediated through drinking motives, with comparable contributions from enhancement and coping motives. Homosexual men, however, displayed similar or lower scores for alcohol use indicators and AUD symptoms than heterosexual men, but these differences were not mediated by drinking motives. Indeed, homosexual men exhibited greater coping motives than heterosexual men. No significant results or discernible patterns emerged for bisexual or mostly-homosexual men.

**Discussion:**

These findings highlight the importance of considering the full spectrum of sexual orientations in healthcare and of broadening the focus on drinking motives beyond coping. Understanding the varied motives for alcohol use across the spectrum of sexual orientations facilitates tailored prevention strategies.

## 1 Introduction

**Figure 1:**
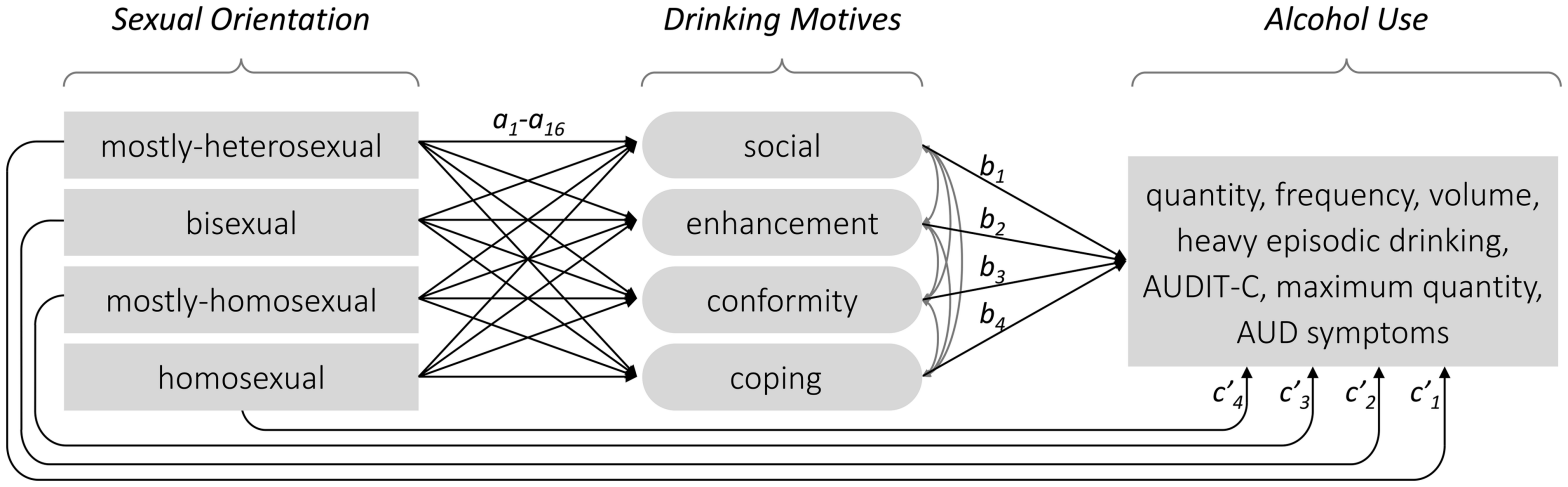
Schematic representation of our structural model for estimating direct pathways (c’) and indirect pathways from sexual orientation (ref = heterosexual participants) through drinking motives (a*b) and to alcohol use. Separate models were estimated for each indicator of alcohol use. All the pathways were also adjusted for age and linguistic region. Sexual orientation was dummy-coded with heterosexual participants as the reference group.

Research has consistently shown that lesbian, gay and bisexual (LGB) individuals are at a greater risk than heterosexual individuals of mental health problems such as the problematic use of alcohol and other substances, depression or suicidality (Blondeel et al., 2016; Kerridge et al., 2017; King et al., 2008; Krueger et al., 2020; McCabe et al., 2019; Meads et al., 2023; Vrangalova and Savin-Williams, 2014). King et al. (2008) conducted a systematic review and meta-analysis that estimated a 2.2 times greater risk of alcohol dependence among LGB individuals than among heterosexual individuals. More recent studies have provided new evidence indicating significant substance use disparities across the spectrum of sexual orientation. In public health research, sexual orientation is often categorized into simplified groups, such as heterosexual, bisexual and homosexual—sometimes just heterosexual and sexual minority—disregarding important distinctions (Savin-Williams, 2016). Several authors (Blum et al., 2019; Parnes et al., 2017; Wicki et al., 2021) have noted that important differences that may exist along the spectrum of sexual orientation (Kinsey et al., 1948) are thereby obscured. Studies have indicated that bisexual individuals have a greater risk of problematic substance use and alcohol use disorder (AUD) than their heterosexual or homosexual peers (Demant et al., 2016; Schuler and Collins, 2020; Schulz et al., 2022a). Notably, research by Vrangalova and Savin-Williams (2014) observed that the highest risk was among mostly-heterosexual women and men—a finding that was replicated in a large, representative sample of young Swiss men (Wicki et al., 2021). The present study aimed to investigate whether drinking motives (DMs) mediated the associations between sexual orientation and indicators of alcohol use (Figure 1). DMs—the reasons why alcohol is consumed—are conceptualized as gateways through which more distal influences are mediated (Cooper, 1994). A better understanding of the reasons underlying these alcohol consumption patterns would better guide the development of interventions and informed public health strategies.

When trying to explain health disparities across the spectrum of sexual orientation, the *minority stress theory* developed by Meyer (2003) is one of the primary frameworks. According to this theory, health disparities are a result of societal or self-imposed stigma and shame (Dowshen and Ford, 2019; Hatzenbuehler, 2009; Hatzenbuehler et al., 2011; Meyer, 2003). Higher rates of distal stressors (e.g., violence or discrimination) or proximal stressors (e.g., concealment of one’s sexual orientation, internalized stigma or expected discrimination) can account for these disparities (Meyer, 2003). In addition to minority stress, bisexual individuals may also experience *bi-negativity*—negative stereotyping related to bisexuality—such as being seen as indecisive or promiscuous or facing discrimination due to challenging the binary conceptualisation of sexual orientation as either heterosexual or homosexual (Bostwick and Hequembourg, 2014; Mulick and Wright, 2002; Obradors-Campos, 2011; Ochs, 2011). Moreover, bisexual individuals report having fewer role models to rely on and feeling less connected to the Lesbian, Gay, Bisexual, Transgender, Intersex and Queer (LGBTIQ*) community than homosexual individuals (Balsam and Mohr, 2007; Chan et al., 2020).

In addition to the minority stress theory and bi-negativity, higher levels of alcohol use among LGB individuals have also been attributed to cultural factors, such as reliance on socializing in bars and other heavy-drinking venues (Bloomfield et al., 2011; Coulter et al., 2019; Parks and Heller, 2013; Trocki et al., 2009) and established social drinking norms and expectations (Boyle et al., 2017; Boyle et al., 2020). Moreover, other factors correlating with sexual orientation, such as personality traits (e.g., openness and neuroticism; Allen and Walter, 2018; Wang et al., 2014) or a clubbing lifestyle(e.g., clubbing; Trocki et al., 2009) have been shown to increase substance use, either independently of minority stress or biphobia or by mediating or moderating their influence.

According to the *motivational model of alcohol use* (MMAU; Cox and Klinger, 1988, 1990), DMs represent the final step in the decision-making process of whether or not to drink alcohol. This model states that DMs mediate the effects of distal factors (e.g., sex, age, history) and proximal factors (e.g., drinking expectations and the immediate context) (Dworkin et al., 2018; Kuntsche et al., 2015; Martin et al., 2019; Van Damme et al., 2015). As DMs are the most proximal factors for alcohol use, researchers have argued that motives are more helpful to prevention efforts than distal factors (Bresin and Mekawi, 2021; Canale et al., 2015; Conrod et al., 2013; Cooper, 1994; Cooper et al., 2016; Cox and Klinger, 1988; Lammers et al., 2017; Newton et al., 2016; Wurdak et al., 2016). DMs can be characterized by two distinct dimensions that describe whether (1) alcohol use is motivated by a desire to achieve a positive state or to avoid negative states, and (2) whether the focus is internal (directed toward alcohol’s pharmacological effects) or external (directed toward social outcomes). The Drinking Motives Questionnaire combines and builds on these two dimensions to yield four distinct DMs (Cooper, 1994): social (positive, external: ‘because it makes social gatherings more fun’); enhancement (positive, internal: ‘because I like the feeling’); coping (negative, internal: ‘to forget about my problems’) and conformity (negative, external: ‘so I will not feel left out’). Large cross-cultural studies have consistently found that social DMs are reported most, followed by enhancement, coping and, lastly, conformity DMs (Bresin and Mekawi, 2021; Cooper, 1994; Wicki et al., 2017). When considering zero-order correlations, all four kinds of DMs show positive associations with each other and with other indicators of alcohol use (Bresin and Mekawi, 2021; Cooper, 1994; Wicki et al., 2017). However, when DMs are analyzed simultaneously and across studies, social DMs are positively associated with frequent yet moderate drinking. Indeed, the two internal DMs (enhancement and coping) are positively associated with high daily average alcohol use and heavy episodic drinking, whereas conformity DMs are negatively associated with alcohol use when all three other DM dimensions are considered (for an overview see Bresin and Mekawi, 2021; Cooper et al., 2016). Due to this considerable covariance between DMs and to fully understand the motivational pathways for alcohol due to internal versus external and to positive versus negative reinforcement, it is important to consider all four DMs simultaneously.

Empirical studies exploring differences in DMs across the spectrum of sexual orientation are rare and often limited to specific segments of the spectrum or subsets of the sexual minority. Existing studies have yielded inconsistent findings, with one study indicating higher rates of coping and conformity DMs among non-heterosexual than among heterosexual adolescents (Bos et al., 2016), while other studies reported no significant differences in DMs between bisexual and homosexual individuals (Boyle et al., 2017; Fairlie et al., 2018; Ristuccia et al., 2019). To the best of our knowledge, only one study has directly explored the mediating role of DMs in the relationship between sexual orientation and alcohol use. Bos et al. (2016) found higher weekday alcohol use among non-heterosexual individuals than among heterosexual individuals; however, neither coping nor conformity DMs mediated this association.

To understand the pathways between aspects of minority stress or bi-negativity and alcohol use patterns, several studies have explored the mediating effects of DMs. Best documented is the mediating role of drinking to cope, such as for bi-negativity (Schulz et al., 2022b), discrimination due to sexual orientation (Hatzenbuehler et al., 2011; Lewis et al., 2016; Wray et al., 2016) or discrimination due to a variety of minority-status-based discrimination (e.g., due to ethnic minority or LGB; Hatzenbuehler et al., 2011), post-traumatic stress (Dworkin et al., 2021), stigma-related stress (Lewis et al., 2017), internalized stigma (Feinstein and Newcomb, 2016; Matsuzaka et al., 2023), sexual orientation self-concept ambiguity (Hancock et al., 2018) or sexual coercion among bisexual women (Kelley et al., 2018). Other DMs mediating pathways to alcohol use are rarely tested: enhancement DMs have been shown to mediate the association with discrimination due to sexual orientation (Wray et al., 2016) or internalized stigma (Feinstein and Newcomb, 2016), and conformity DMs have been shown to mediate the associations with stigma-related stress (Lewis et al., 2017).

The preponderance of studies examining coping DMs to elucidate the pathways linking aspects of minority stress or bi-negativity with alcohol use can be explained as they take the self-medication hypothesis (Khantzian, 1997) as a rationale. According to this hypothesis, individuals use alcohol as an avoidant coping mechanism when confronted with psychological symptoms or subjective distress (Khantzian, 1997). However, as Bresin and Mekawi (2021) pointed out, while avoidance or coping DMs may seem to be direct ways of reacting to such stressors, some individuals may also seek to enhance their pleasant feelings in response to stress and negative affect (Waugh, 2020; Waugh et al., 2020). Indeed, coping and enhancement motives reflect general tendencies of avoidance versus approach (Cooper et al., 2016). Additionally, they are specifically linked to alcohol use (Bresin and Mekawi, 2021). For an understanding of aetiological pathways and how to implement interventions, it is crucial to understand whether alcohol consumption is driven by coping DMs, indicative of an avoidance orientation, or by enhancement DMs, indicative of an approach orientation. For coping DMs, interventions should focus on developing skills to confront stressors as merely avoiding them can exacerbate the problem; conversely, for enhancement DMs, it is essential to explore and educate individuals about alternate sources of enhancement than alcohol (Bresin and Mekawi, 2021). Thus, to understand the pathways between stressors and alcohol use it is important to consider all four DMs, not just coping (Dworkin et al., 2021).

The present study was based on a large sample of young Swiss men and considered several shortcomings in the literature when exploring whether DMs mediated associations between the spectrum of sexual orientations and alcohol use (Figure 1). We began our analysis by considering all four DMs according to the motivational model of alcohol use (Cox and Klinger, 1988, 1990) to enable a more comprehensive understanding of the motivational pathways to alcohol use and AUD symptoms (Bresin and Mekawi, 2021). Second, we used a five-point sexual orientation scale to avoid blurring potential associations by aggregating groups (e.g., Savin-Williams, 2016; Vrangalova and Savin-Williams, 2014) or limiting our scope to a binary choice between non-heterosexual and heterosexual participants (e.g., Boyle et al., 2017; Fairlie et al., 2018; Ristuccia et al., 2019). Third, the study used a non-self-selective, general population sample to minimize potential bias (Salway et al., 2019). Previous studies relied on self-selection through online platforms such as Facebook or Craigslist (Boyle et al., 2017; Fairlie et al., 2018; Feinstein and Newcomb, 2016; Ristuccia et al., 2019; Wray et al., 2016) or school or university samples (Bos et al., 2016; Hatzenbuehler et al., 2011). Fourth, the present study looked at young Swiss men. A large proportion of the research on DMs (Fairlie et al., 2018; Feinstein and Newcomb, 2016; Hancock et al., 2018; Hatzenbuehler et al., 2011; Kelley et al., 2018; Lewis et al., 2016; Ristuccia et al., 2019; Wray et al., 2016) or on problematic substance use (King et al., 2008; Plöderl and Tremblay, 2015) among LGBs has been based on samples from the United States. This is important, as the rare cross-cultural research on alcohol use in sexual minority groups (Bloomfield et al., 2011) indicates that findings from the USA cannot be generalized to other countries. For instance, in several countries across Europe, Latin America, North America, and Australasia, individuals in same-sex relationships had no greater risk of being heavy drinkers or engaging in risky drinking compared to those in mixed-gender relationships, with the exception of lesbians in North America, who showed a greater risk for high-volume drinking and heavy episodic drinking (Bloomfield et al., 2011).

Based on the minority stress theory (Meyer, 2003) and bi-negativity (Bostwick and Hequembourg, 2014; Mulick and Wright, 2002; Obradors-Campos, 2011; Ochs, 2011), we hypothesized that the higher levels of alcohol use seen in certain subgroups of the sexual orientation spectrum were mediated by greater coping and enhancement DMs. Drinking occurs to reduce or counteract negative affect due to minority stress and/or bi-negativity. In accordance with studies on peer substance use norms (e.g., Boyle et al., 2017), we hypothesized that conformity and social decision-making also mediated relationships between sexual orientation and alcohol use. Drinking occurs to avoid negative reactions or achieve positive reactions by corresponding to the norm. However, as this was the first study to explore whether DMs mediate pathways between sexual orientation and alcohol use and to consider all four DMs simultaneously, no hypotheses about the relative strengths of their mediation effects were formulated.

## 2 Materials and methods

### 2.1 Sample

The present study used data from the third wave of the Cohort Study on Substance Use Risk Factors (C-SURF). C-SURF capitalized on a unique opportunity by enrolling a representative sample of young Swiss men from 21 of Switzerland’s 26 cantons. Military service is mandatory for all Swiss men at the age of 19, requiring them to report to report to designated recruitment centers for an assessment of their suitability for military, civil or no service. Between August 2010 and November 2011, all the young men reporting to the centers in Windisch, Mels and Lausanne were informed by the research staff about the cohort study and invited to participate. Of the 13,245 individuals who were approached by the research staff, 7,556 (57.0%) provided written informed consent. Within 2 weeks after the suitability assessment, those who had consented were invited to participate in the first wave of data collection. It is important to note that the recruitment centers were solely used for the purpose of informing and enrolling participants; the study itself was entirely independent of the military, and participants completed their questionnaires at home and not while actively in military service. General-purpose gift vouchers were provided as incentives to participate. Notably, study participants and non-participants were very similar in terms of their alcohol, cigarette and cannabis use patterns and levels of education and urban or rural inhabitants (Studer et al., 2013a). The Human Research Ethics Committee of the Canton of Vaud approved the study. Additional comprehensive information regarding C-SURF has been reported previously (Gmel et al., 2015; Gmel et al., 2021; Studer et al., 2013a; Studer et al., 2013b).^1^

A total of 5,516 men participated in the present study based on C-SURF’s third wave, representing 73.0% of those who gave informed consent at baseline (participation rate for the third wave) and 41.6% of those initially approached by the research staff (overall participation rate). Data were collected between April 2016 and March 2018. Participants who reported no alcohol use in the 12 months prior to the study (*n* = 370, 6.7%) or who chose not to answer the sexual attraction question (*n* = 7, < 0.1%) were excluded from the analysis. To explore potential differences in 12-month abstinence across sexual orientation groups, abstinence was regressed in a logistic model on dummy-coded sexual orientation, with heterosexual men (the largest group) as the reference category. While abstinence rates among mostly-heterosexual (5.0%), bisexual (5.4%), and homosexual men (7.9%) did not differ significantly from heterosexual men (6.7%), abstinence was significantly higher among mostly-homosexual men (19.4%; b = 1.21, *p* = 0.004). The final analytical sample consisted of 5,139 men. Participants’ mean age was 25.4 years old (SD = 1.24), with 57.5% (*n* = 3,172) from French-speaking Switzerland and 42.5% (*n* = 2,344) from German-speaking Switzerland.

### 2.2 Measures

#### 2.2.1 Criterion variables

*Alcohol use indicators*. The frequency of alcohol use per year and usual quantities consumed were calculated using four questions about participants’ usual alcohol use on weekdays and weekends (over the past 12 months). These variables were used to calculate usual volumes and numbers of drinks per week. The questionnaire showed an illustration of the equivalents of standard drinks (corresponding to 10 g of pure alcohol). Heavy episodic drinking (HED) was assessed by asking how often participants had drunk six or more standard drinks on one occasion in the past 12 months, with the five answer options ranging from ‘never’ to ‘every day or almost every day’ coded as occasions per year. The Alcohol Use Disorders Identification Test-Consumption (AUDIT-C) score, a measure of hazardous and harmful alcohol use, was calculated based on indicators of alcohol use frequency, quantity, and HED (Aalto et al., 2009; Bush et al., 1998; Duffy et al., 2023). Consumption maximums were based on an open-ended question asking about the largest number of standard alcoholic drinks participants had drunk in a single day over the past 12 months (Greenfield et al., 2006).

*AUD symptoms*. Self-reported AUD symptoms were based on a questionnaire (Knight et al., 2002) adapted from the Semi-Structured Assessment for the Genetics of Alcoholism (SSAGA; Bucholz et al., 1994; Hesselbrock et al., 1999) and assessed 11 symptoms of AUD over the past 12 months.

#### 2.2.2 Predictor and mediator variables

*Sexual orientation*. Using the Reduced Kinsey Scale (Bailey et al., 2016), which focuses specifically on the dimension of sexual attraction (Patterson et al., 2017), participants were asked whether they felt sexually attracted to ‘women only’ (labeled as heterosexual), ‘women predominantly’ (mostly-heterosexual), ‘both women and men equally’ (bisexual), ‘men predominantly’ (mostly-homosexual) and ‘men only’ (homosexual). Four dummy-coded variables were created with ‘heterosexual’ as the reference category.

*Drinking motives*. The Drinking Motives Questionnaire–Revised–Short Form (DMQ-R SF; Kuntsche and Kuntsche, 2009) was used to assess social DMs (e.g., ‘because it makes social gatherings more fun’), enhancement DMs (e.g., ‘because I like the feeling’), coping DMs (e.g., ‘because it helps when I feel depressed or nervous’) and conformity DMs (e.g., ‘so I will not feel left out’). For each of the questionnaire’s 12 items, participants were asked to rate how often they had consumed alcohol over the past year. On a five-point scale, answers ranged from (almost) never (coded as 1) to (almost) always (coded as 5) (Cronbach’s *α* varied between 0.806 and 0.859).

*Sociodemographic variables*. Participants’ linguistic region (coded as 0 and 1 for French-speaking and Germans-speaking, respectively) and age were used for adjustment.

### 2.3 Statistical analysis

Descriptive statistics were calculated using STATA 16.0 software (StataCorp, 2017). All other analyses were performed using Mplus, version 8 (Muthén and Muthén, 1998/2017), using a full information maximum likelihood (FIML) approach to include cases with missing values. Structural equation models were used to test whether possible associations between sexual orientation and indicators of alcohol use were mediated by DMs (Figure 1). Separate structural equation models were estimated for each indicator of alcohol use (quantity, frequency, volume, HED, maximum and AUDS) based on linear or ordinal probit regression models. All pathways were also adjusted for age and linguistic region. Sexual orientation was recoded into four dummy variables, using the largest subgroup (labeled ‘heterosexual’) as the reference group. DMs were treated as latent variables, with each based on three items. As the distributions of the indicators on alcohol use quantity, frequency, volume and maximum drinks were right-skewed, a logarithmic transformation was applied. HED was treated as an ordinal variable, and overall AUD symptoms were treated as a latent variable based on 11 symptoms.

The structural equation models were estimated using the delta method to obtain standard errors for the indirect mediated pathways (a*b paths in Figure 1), and 1,000 bootstrap replications were used to reduce the impact of non-normality and outliers (Biesanz et al., 2010; Shrout and Bolger, 2002). The sum of direct associations and the indirect mediated associations yielded the total association [e.g., for mostly-heterosexual individuals: $c=c\prime_{1}+\overset{4}{\underset{i=1}{\sum }}\left(a_{i}*b_{i}\right)$ ]. Non-standardized coefficients, standardized *β*-coefficients and their 95% confidence intervals were reported to allow the direct comparison of pathways across outcome variables and to quantify uncertainty (Muthén and Muthén, 1998/2017). The comparative fit index (CFI), the Tucker–Lewis index (TLI) and the root mean square error of approximation (RMSEA) were used to examine the model’s fit. Indicators of a good fit are a CFI and a TLI > 0.95 and an RMSEA ≤0.06. However, a CFI and a TLI > 0.90 and an RMSEA ≤0.08 are generally also considered acceptable (Brown, 1993; Hu and Bentler, 1999; Kline, 2023).

## 3 Results

When questioned about their sexual orientation (as sexual attraction on the Reduced Kinsey Scale), 89.3% of participants reported being attracted to ‘women only’, 6.7% to ‘women predominantly’, 1.0% to ‘both men and women’, 0.6% to ‘men predominantly’ and 2.3% to ‘men only’ (Table 1). In our analytical sample of 5,319 non-abstainers, the average participant drank alcoholic beverages 94.0 days per year and consumed 3.8 standard drinks on a usual drinking day, corresponding to a volume of 7.7 standard drinks per week. Regarding AUDIT-C scores, the percentage of participants screened positive for hazardous and harmful alcohol use varied depending on the chosen cut-off: 73.1% with a cut-off of 4+, 41.6% with 6+, and 17.5% with 8+. The frequency of HED (i.e., six or more standard drinks on an occasion or ≥ 60 g of pure alcohol) was 18.0 days per year and, on average, alcohol consumers’ maximum number of drinks was 10.4. Based on self-assessment, 34.1% of the sample met the criteria for mild AUD (i.e., at least two symptoms), 9.5% for moderate AUD (i.e., at least four symptoms), and 2.4% for severe AUD (i.e., at least six symptoms). Zero-order correlations between DMs, alcohol use indicators and AUDS were all positive except for the conformity DMs that were not associated with maximum quantity. For the main analysis, the fit indices generally signaled a good or acceptable fit for the structural equation models predicting criterion variables (see Supplementary Table S1 for details).

**Table 1:** Description of the study sample (among non-abstainers only) and correlations between sexual orientation, drinking motives and alcohol use indicators.

	*n*	%/*M* (SD)		Sexual orientation					Drinking motives				Alcohol use					
				1	2	3	4	5	6	7	8	9	10	11	12	13	14	15
Sexual orientation																		
(1) Heterosexual	4,521	89.3%																
(2) Mostly-heterosexual	341	6.7%		---														
(3) Bisexual	53	1.1%		---	---													
(4) Mostly-homosexual	29	0.6%		---	---	---												
(5) Homosexual	117	2.3%		---	---	---	---											
Drinking motives																		
(6) Social	5,060	2.85	(1.04)	0.002	0.017	**−0.032**	0.009	−0.015										
(7) Enhancement	5,062	2.76	(1.03)	**−0.040**	**0.059**	−0.013	−0.001	−0.007	**0.710**									
(8) Coping	5,060	1.69	(0.81)	**−0.069**	**0.054**	0.021	0.001	**0.036**	**0.357**	**0.363**								
(9) Conformity	5,061	1.35	(0.63)	**−0.058**	**0.056**	0.003	0.013	0.018	**0.306**	**0.210**	**0.364**							
Alcohol use																		
(10) Quantity	5,138	3.76	(2.36)	−0.002	0.026	0.006	0.005	**−0.045**	**0.424**	**0.469**	**0.232**	**0.075**						
(11) Frequency	5,138	94.01	(76.97)	**−0.033**	**0.040**	0.012	−0.006	−0.005	**0.324**	**0.371**	**0.262**	**0.076**	**0.372**					
(12) Volume	5,138	7.73	(9.40)	−0.026	**0.041**	0.011	−0.003	−0.022	**0.426**	**0.481**	**0.298**	**0.090**	**0.707**	**0.920**				
(13) HED	5,135	17.96	(41.89)	−0.021	**0.041**	0.000	0.005	**−0.028**	**0.352**	**0.406**	**0.237**	**0.099**	**0.522**	**0.495**	**0.621**			
(14) AUDIT-C	5,138	5.09	(2.41)	−0.021	**0.043**	0.004	−0.001	**−0.031**	**0.477**	**0.531**	**0.287**	**0.092**	**0.767**	**0.784**	**0.922**	**0.810**		
(15) Maximum	5,126	10.39	(7.40)	−0.005	**0.036**	−0.013	−0.011	**−0.037**	**0.401**	**0.471**	**0.165**	0.009	**0.565**	**0.495**	**0.616**	**0.588**	**0.684**	
(16) AUDS	5,130	1.33	(1.63)	**−0.061**	**0.075**	0.014	0.013	−0.016	**0.419**	**0.459**	**0.350**	**0.153**	**0.417**	**0.455**	**0.523**	**0.442**	**0.518**	**0.445**

Correlations in bold are significant at *p* < 0.05; all correlations are Pearson’s correlation coefficients, except for Kendall’s tau for ‘maximum no’. Sexual orientation = dummy-coded sexual orientation (e.g., for ‘heterosexual’: heterosexual = 1, all other options = 0); HED = heavy episodic drinking; AUDIT-C = Alcohol Use Disorders Identification Test-Consumption score; AUDS = alcohol use disorder symptoms. For descriptive statistics, the M (SD) for drinking motives and AUDS were based on the mean scores of their respective items. HED was transformed into days per year, and values before log-transformation are reported. --- = non-reported between-dummy-coded variable correlations.

Coefficients for total, direct and total indirect associations are presented in Table 2. Depending on the criterion variable, the R^2^ varied between 0.082 for quantity and 0.363 for AUDIT-C. Mostly-heterosexual men reported significantly higher volumes, more HED, higher AUDIT-C scores, higher maximums and more severe AUD than heterosexual men (see Supplementary Table S2 for details). All the significant pathways were positive for mostly-heterosexual men, and two different patterns of mediation were found. First, full (or almost full) mediation through DMs was found for quantities, frequencies, volumes, HED, AUDIT-C scores, and maximums, as indicated by significant total indirect associations and non-significant direct associations. Second, partial mediation was found for AUD symptoms (with about half of that association mediated through DMs). For bisexual and mostly-homosexual men, none of the associations were significant, possibly due to small sample sizes. For homosexual men, the total associations were generally negative, but they were significant for four indicators and only consisted of direct effects for quantity, frequency, HED and AUDIT-C scores.

**Table 2:** Total, direct and total indirect associations (mediated through drinking motives) between sexual orientation and indicators of alcohol use.

		Total indirect association			Direct association			Total indirect association		
		*b*	*β*	*β_95%CI_*	*b*	*β*	*β_95%CI_*	*b*	*β*	*β_95%CI_*
Quantity (*R*^2^ = 0.082)										
Mostly-heterosexual	l	0.028	0.019	[−0.009; 0.052]	0.000	0.000	[−0.029; 0.031]	**0.027**	**0.019**	**[0.008; 0.030]**
Bisexual	l	−0.020	−0.006	[−0.030; 0.020]	−0.016	−0.005	[−0.028; 0.021]	−0.004	−0.001	[−0.011; 0.008]
Mostly-homosexual	l	−0.019	−0.004	[−0.028; 0.026]	−0.011	−0.002	[−0.027; 0.028]	−0.008	−0.002	[−0.009; 0.007]
Homosexual	l	**−0.091**	**−0.038**	**[−0.054; −0.018]**	**−0.091**	**−0.038**	**[−0.056; −0.017]**	0.000	0.000	[−0.010; 0.009]
Frequency (*R*^2^ = 0.280)										
Mostly-heterosexual	l	0.055	0.023	[−0.006; 0.051]	−0.011	−0.005	[−0.029; 0.019]	**0.067**	**0.028**	**[0.010; 0.046]**
Bisexual	l	0.032	0.006	[−0.019; 0.029]	0.076	0.013	[−0.009; 0.034]	−0.043	−0.007	[−0.025; 0.009]
Mostly-homosexual	l	0.025	0.003	[−0.024; 0.028]	0.036	0.005	[−0.017; 0.024]	−0.011	−0.001	[−0.015; 0.014]
Homosexual	l	**−0.172**	**−0.044**	**[−0.068; −0.019]**	**−0.158**	**−0.040**	**[−0.063; −0.015]**	−0.014	−0.003	[−0.020; 0.012]
Volume (*R*^2^ = 0.197)										
Mostly-heterosexual	l	**0.172**	**0.040**	**[0.011; 0.066]**	0.056	0.013	[−0.012; 0.036]	**0.116**	**0.027**	**[0.011; 0.042]**
Bisexual	l	0.141	0.013	[−0.017; 0.040]	0.149	0.014	[−0.010; 0.037]	−0.008	−0.001	[−0.016; 0.013]
Mostly-homosexual	l	−0.115	−0.008	[−0.040; 0.020]	−0.096	−0.007	[−0.035; 0.019]	−0.019	−0.001	[−0.013; 0.011]
Homosexual	l	−0.021	−0.003	[−0.031; 0.023]	−0.048	−0.007	[−0.033; 0.018]	0.027	0.004	[−0.011; 0.017]
HED (*R*^2^ = 0.359)										
Mostly-heterosexual	op	**0.182**	**0.045**	**[0.013; 0.093]**	0.041	0.010	[−0.018; 0.046]	**0.141**	**0.035**	**[0.017; 0.063]**
Bisexual	op	0.064	0.006	[−0.028; 0.039]	0.123	0.012	[−0.015; 0.038]	−0.060	−0.006	[−0.025; 0.012]
Mostly-homosexual	op	0.009	0.001	[−0.027; 0.024]	0.037	0.003	[−0.020; 0.022]	−0.028	−0.002	[−0.017; 0.014]
Homosexual	op	**−0.250**	**−0.037**	**[−0.084; −0.025]**	**−0.230**	**−0.034**	**[−0.063; −0.018]**	−0.020	−0.003	[−0.033; 0.009]
AUDIT-C (*R*^2^ = 0.363)										
Mostly-heterosexual	l	**0.096**	**0.041**	**[0.015; 0.067]**	0.019	0.008	[−0.013; 0.029]	**0.077**	**0.033**	**[0.012; 0.052]**
Bisexual	l	0.026	0.004	[−0.024; 0.032]	0.064	0.011	[−0.010; 0.032]	−0.039	−0.007	[−0.026; 0.012]
Mostly-homosexual	l	−0.028	−0.004	[−0.033; 0.023]	−0.016	−0.002	[−0.026; 0.019]	−0.012	−0.002	[−0.017; 0.015]
Homosexual	l	**−0.115**	**−0.030**	**[−0.058; −0.005]**	**−0.109**	**−0.028**	**[−0.053; −0.005]**	−0.006	−0.002	[−0.020; 0.016]
Maximum (*R*^2^ = 0.308)										
Mostly-heterosexual	l	**0.228**	**0.041**	**[0.012; 0.067]**	0.046	0.008	[−0.015; 0.029]	**0.182**	**0.033**	**[0.014; 0.051]**
Bisexual	l	0.173	0.013	[−0.019; 0.039]	0.226	0.016	[−0.006; 0.038]	−0.052	−0.004	[−0.022; 0.014]
Mostly-homosexual	l	−0.091	−0.005	[−0.035; 0.024]	−0.061	−0.003	[−0.028; 0.020]	−0.029	−0.002	[−0.016; 0.014]
Homosexual	l	−0.192	−0.021	[−0.047; 0.005]	−0.206	−0.022	[−0.047; 0.002]	0.014	0.001	[−0.016; 0.017]
AUDS (*R*^2^ = 0.336)										
Mostly-heterosexual	l	**0.053**	**0.086**	**[0.047; 0.125]**	**0.026**	**0.043**	**[0.011; 0.076]**	**0.026**	**0.043**	**[0.023; 0.063]**
Bisexual	l	0.056	0.037	[−0.010; 0.088]	0.047	0.031	[−0.005; 0.071]	0.009	0.006	[−0.014; 0.025]
Mostly-homosexual	l	0.042	0.021	[−0.011; 0.056]	0.041	0.020	[−0.003; 0.045]	0.001	0.001	[−0.014; 0.016]
Homosexual	l	−0.001	−0.001	[−0.034; 0.032]	−0.016	−0.016	[−0.044; 0.015]	0.016	0.015	[−0.002; 0.033]

Coefficients in bold are significant at *p* < 0.05; HED = heavy episodic drinking; AUDIT-C = Alcohol Use Disorders Identification Test-Consumption score; AUDS = alcohol use disorder symptoms; mostly-heterosexual/…/homosexual = dummy-coded sexual minority (reference = heterosexual); l/op = linear/ordinal probit regression model; b = unstandardized coefficient of association; β = standardized coefficient of association; 95% CI = 95% bootstrap confidence interval. Total association = direct + total indirect associations; direct associations = c’ paths; total indirect associations = sum of specific a*b paths through drinking motives.

The associations between the dummy-coded sexual orientation variable and DMs (a paths, Table 3) indicated that mostly-heterosexual men had higher rates of enhancement, coping and conformity DMs than heterosexual men. Lower rates of social DMs were found among bisexual men, and higher rates of coping DMs were found among homosexual men. No clear patterns could be identified for mostly-homosexual men; all their coefficients were non-significant and had small effect sizes. Looking at the b paths, which indicated associations between DMs and indicators of alcohol use, the associations were strongest with enhancement DMs, while being considerably weaker for coping and conformity DMs. However, associations between AUD symptoms and the enhancement and coping DMs were comparably strong and positive.

**Table 3:** Associations between sexual orientation and drinking motives (a paths) and between drinking motives and indicators of alcohol use (b paths).

		Social DM			Enhancement DM			Coping DM			Conformity DM		
		*b*	*β*	*β_95%CI_*	*b*	*β*	*β_95%CI_*	*b*	*β*	*β_95%CI_*	*b*	*β*	*β_95%CI_*
SO→DM (a paths)													
Mostly-heterosexual	l	0.056	0.015	[−0.016; 0.044]	**0.250**	**0.064**	**[0.029; 0.095]**	**0.189**	**0.059**	**[0.027; 0.091]**	**0.132**	**0.062**	**[0.027; 0.096]**
Bisexual	l	**−0.270**	**−0.030**	**[−0.063; −0.001]**	−0.119	−0.012	[−0.045; 0.020]	0.217	0.027	[−0.007; 0.060]	0.040	0.008	[−0.023; 0.042]
Mostly-homosexual	l	0.068	0.006	[−0.024; 0.033]	−0.028	−0.002	[−0.027; 0.025]	0.030	0.003	[−0.019; 0.026]	0.088	0.012	[−0.014; 0.047]
Homosexual	l	−0.098	−0.016	[−0.044; 0.009]	−0.036	−0.005	[−0.036; 0.023]	**0.254**	**0.047**	**[0.016; 0.080]**	0.079	0.022	[−0.007; 0.055]
DM→alcohol use (b paths)													
Quantity	l	−0.029	−0.072	[−0.156; 0.008]	**0.121**	**0.329**	**[0.239; 0.411]**	**0.018**	**0.041**	**[0.001; 0.081]**	**−0.035**	**−0.053**	**[−0.090; −0.015]**
Frequency	l	**0.052**	**0.080**	**[0.003; 0.152]**	**0.277**	**0.453**	**[0.381; 0.535]**	**0.032**	**0.044**	**[0.007; 0.080]**	**−0.084**	**−0.076**	**[−0.112; −0.041]**
Volume	l	0.003	0.003	[−0.076; 0.080]	**0.390**	**0.358**	**[0.283; 0.433]**	**0.216**	**0.163**	**[0.129; 0.196]**	**−0.169**	**−0.085**	**[−0.119; −0.052]**
HED	op	0.018	0.016	[−0.067; 0.089]	**0.570**	**0.553**	**[0.481; 0.633]**	**0.118**	**0.090**	**[0.052; 0.128]**	**−0.138**	**−0.079**	**[−0.114; −0.046]**
AUDIT-C	l	**0.048**	**0.074**	**[0.004; 0.142]**	**0.302**	**0.505**	**[0.439; 0.578]**	**0.070**	**0.098**	**[0.069; 0.129]**	**−0.107**	**−0.099**	**[−0.131; −0.068]**
Maximum	l	0.059	0.038	[−0.037; 0.105]	**0.661**	**0.461**	**[0.392; 0.535]**	**0.249**	**0.144**	**[0.112; 0.177]**	**−0.253**	**−0.097**	**[−0.129; −0.064]**
AUDS	l	−0.003	−0.019	[−0.110; 0.064]	**0.052**	**0.331**	**[0.245; 0.418]**	**0.065**	**0.342**	**[0.293; 0.392]**	**0.011**	**0.038**	**[−0.027; 0.104]**

Coefficients in bold are significant at *p* < 0.05; SO = sexual orientation, DM = drinking motive; mostly-heterosexual/…/homosexual = dummy-coded sexual minority (reference = heterosexual); HED = heavy episodic drinking; AUDIT-C = Alcohol Use Disorders Identification Test-Consumption score; AUDS = alcohol use disorder symptoms; l/op = linear/ordinal probit regression model; b = unstandardized coefficient of association; β = standardized coefficient of association; 95% CI = 95% bootstrap confidence interval.

Specific indirect associations (a*b paths) are presented in Table 4. For mostly-heterosexual men, the positive and significant total indirect associations with alcohol use indicators were due to the indirect effects through enhancement DMs. Positive indirect effects through coping DMs were considerably smaller (and non-significant for quantity), and small but negative (i.e., protective indirect) associations were found through conformity DMs. However, for AUD symptoms, the indirect associations through enhancement and coping DMs were positive and of similar strength, while no indirect associations through conformity DMs were found. For bisexual and mostly-homosexual participants, no significant indirect associations or clear patterns were found. For homosexual men, the total indirect effects were not significant. Nonetheless, for all the indicators of alcohol use, their significant positive associations through coping DMs were counterbalanced by non-significant indirect associations through the other DMs.

**Table 4:** Specific indirect associations (mediated through specific drinking motives) between sexual orientation and indicators of alcohol use (a*b paths).

		Social DM			Enhancement DM			Coping DM			Conformity DM		
		*b*	*β*	*β_95%CI_*	*b*	*β*	*β_95%CI_*	*b*	*β*	*β_95%CI_*	*b*	*β*	*β_95%CI_*
Quantity													
Mostly-heterosexual	l	−0.002	−0.001	[−0.005; 0.001]	**0.030**	**0.021**	**[0.009; 0.034]**	0.003	0.002	[0.000; 0.005]	**−0.005**	**−0.003**	**[−0.007; −0.001]**
Bisexual	l	0.008	0.002	[−0.001; 0.007]	−0.014	−0.004	[−0.015; 0.007]	0.004	0.001	[0.000; 0.003]	−0.001	0.000	[−0.002; 0.001]
Mostly-homosexual	l	−0.002	0.000	[−0.003; 0.002]	−0.003	−0.001	[−0.009; 0.008]	0.001	0.000	[−0.001; 0.001]	−0.003	−0.001	[−0.003; 0.001]
Homosexual	l	0.003	0.001	[−0.001; 0.005]	−0.004	−0.002	[−0.012; 0.008]	0.005	0.002	[0.000; 0.005]	−0.003	−0.001	[−0.004; 0.000]
Frequency													
Mostly-heterosexual	l	0.003	0.001	[−0.002; 0.004]	**0.069**	**0.029**	**[0.013; 0.046]**	0.006	0.003	[0.000; 0.005]	**−0.011**	**−0.005**	**[−0.009; −0.002]**
Bisexual	l	−0.014	−0.002	[−0.007; 0.000]	−0.033	−0.006	[−0.021; 0.009]	0.007	0.001	[0.000; 0.003]	−0.003	−0.001	[−0.004; 0.002]
Mostly-homosexual	l	0.003	0.000	[−0.002; 0.003]	−0.008	−0.001	[−0.012; 0.011]	0.001	0.000	[−0.001; 0.001]	−0.007	−0.001	[−0.004; 0.001]
Homosexual	l	−0.005	−0.001	[−0.005; 0.001]	−0.010	−0.003	[−0.016; 0.010]	0.008	0.002	[0.000; 0.005]	−0.007	−0.002	[−0.004; 0.001]
Volume													
Mostly-heterosexual	l	0.000	0.000	[−0.002; 0.002]	**0.098**	**0.023**	**[0.010; 0.036]**	**0.041**	**0.010**	**[0.004; 0.016]**	**−0.022**	**−0.005**	**[−0.009; −0.002]**
Bisexual	l	−0.001	0.000	[−0.003; 0.003]	−0.047	−0.005	[−0.017; 0.007]	0.047	0.004	[−0.001; 0.010]	−0.007	−0.001	[−0.004; 0.002]
Mostly-homosexual	l	0.000	0.000	[−0.001; 0.001]	−0.011	−0.001	[−0.010; 0.009]	0.006	0.000	[−0.003; 0.004]	−0.015	−0.001	[−0.004; 0.001]
Homosexual	l	0.000	0.000	[−0.002; 0.002]	−0.014	−0.002	[−0.013; 0.008]	**0.055**	**0.008**	**[0.003; 0.013]**	−0.013	−0.002	[−0.005; 0.001]
HED													
Mostly-heterosexual	op	0.001	0.000	[−0.002; 0.003]	**0.139**	**0.035**	**[0.017; 0.059]**	**0.021**	**0.005**	**[0.002; 0.010]**	**−0.020**	**−0.005**	**[−0.009; −0.002]**
Bisexual	op	−0.005	−0.001	[−0.004; 0.003]	−0.073	−0.007	[−0.025; 0.009]	0.024	0.002	[−0.001; 0.006]	−0.006	−0.001	[−0.004; 0.002]
Mostly-homosexual	op	0.001	0.000	[−0.001; 0.001]	−0.021	−0.002	[−0.015; 0.012]	0.006	0.000	[−0.002; 0.003]	−0.015	−0.001	[−0.004; 0.001]
Homosexual	op	−0.002	0.000	[−0.003; 0.002]	−0.025	−0.004	[−0.032; 0.006]	0.013	0.002	[−0.001; 0.005]	−0.006	−0.001	[−0.005; 0.003]
AUDIT-C													
Mostly-heterosexual	l	0.003	0.001	[−0.001; 0.004]	**0.075**	**0.032**	**[0.015; 0.050]**	**0.013**	**0.006**	**[0.002; 0.010]**	**−0.014**	**−0.006**	**[−0.010; −0.003]**
Bisexual	l	−0.013	−0.002	[−0.006; 0.000]	−0.037	−0.006	[−0.023; 0.010]	0.015	0.003	[−0.001; 0.006]	−0.004	−0.001	[−0.004; 0.002]
Mostly-homosexual	l	0.003	0.000	[−0.002; 0.003]	−0.008	−0.001	[−0.014; 0.012]	0.002	0.000	[−0.002; 0.003]	−0.009	−0.001	[−0.005; 0.001]
Homosexual	l	−0.005	−0.001	[−0.004; 0.001]	−0.011	−0.003	[−0.019; 0.012]	**0.018**	**0.005**	**[0.001; 0.008]**	−0.009	−0.002	[−0.006; 0.001]
Maximum													
Mostly-heterosexual	l	0.003	0.001	[−0.001; 0.003]	**0.165**	**0.030**	**[0.013; 0.045]**	**0.047**	**0.008**	**[0.004; 0.014]**	**−0.034**	**−0.006**	**[−0.010; −0.002]**
Bisexual	l	−0.016	−0.001	[−0.004; 0.001]	−0.080	−0.006	[−0.021; 0.009]	0.054	0.004	[−0.001; 0.009]	−0.010	−0.001	[−0.004; 0.002]
Mostly-homosexual	l	0.004	0.000	[−0.001; 0.002]	−0.018	−0.001	[−0.013; 0.011]	0.007	0.000	[−0.003; 0.004]	−0.022	−0.001	[−0.005; 0.001]
Homosexual	l	−0.006	−0.001	[−0.003; 0.001]	−0.023	−0.003	[−0.017; 0.010]	**0.063**	**0.007**	**[0.002; 0.012]**	−0.020	−0.002	[−0.006; 0.001]
AUDS													
Mostly-heterosexual	l	0.000	0.000	[−0.003; 0.001]	**0.013**	**0.021**	**[0.009; 0.034]**	**0.012**	**0.020**	**[0.009; 0.032]**	0.001	0.002	[−0.002; 0.008]
Bisexual	l	0.001	0.001	[−0.003; 0.004]	−0.006	−0.004	[−0.016; 0.006]	0.014	0.009	[−0.002; 0.021]	0.000	0.000	[−0.001; 0.002]
Mostly-homosexual	l	0.000	0.000	[−0.002; 0.001]	−0.001	−0.001	[−0.009; 0.009]	0.002	0.001	[−0.007; 0.009]	0.001	0.000	[−0.001; 0.003]
Homosexual	l	0.000	0.000	[−0.002; 0.003]	−0.002	−0.002	[−0.012; 0.008]	**0.016**	**0.016**	**[0.005; 0.028]**	0.001	0.001	[−0.001; 0.004]

Coefficients in bold are significant at *p* < 0.05; DM = drinking motives; HED = heavy episodic drinking; AUDIT-C = Alcohol Use Disorders Identification Test-Consumption score; AUDS = alcohol use disorder symptoms; mostly-heterosexual/…/homosexual = dummy-coded sexual minority (reference = heterosexual); l/op = linear/ordinal probit regression model; b = unstandardized coefficient of association; β = standardized coefficient of association; 95% CI = 95% bootstrap confidence interval.

## 4 Discussion

Using a large sample (*N* = 5,139) of young Swiss men, the present study investigated the potential mediating role of drinking motives (DMs) in the relationship between sexual orientation and alcohol use. The vast majority (89.2%) reported being sexually attracted to women only (coded as ‘heterosexual’), with 10.7% of the sample considered to be in sexual minorities. In line with previous research (Vrangalova and Savin-Williams, 2014), we found considerable differences along the spectrum of sexual orientation, with the highest scores on alcohol volume, heavy episodic drinking (HED), Alcohol Use Disorders Identification Test-Consumption (AUDIT-C) scores, maximum number of drinks, and alcohol use disorder (AUD) symptoms reported by mostly-heterosexual men, and scores for quantity, frequency and HED were lowest among homosexual men. Mostly-heterosexual men endorsed more enhancement, coping and conformity DMs than did heterosexual men, while homosexual men endorsed more coping DMs (a paths). As the literature has explored the differences in DMs between heterosexual individuals and the combined sexual minority (Bos et al., 2016) or between bisexual and homosexual individuals (Boyle et al., 2017; Fairlie et al., 2018; Ristuccia et al., 2019), the present study’s findings are not directly comparable: we chose to consider the full five-point spectrum scale of sexual orientation to avoid blurring potential associations by aggregating groups. Nonetheless, a study conducted by Schofield et al. (2023) found that bisexual participants reported higher rates of enhancement and coping motives for cannabis use compared to heterosexual participants. Our findings about the links between DMs and indicators of alcohol use (i.e., b paths and *R*^2^) were in line with the literature (for an overview see Bresin and Mekawi, 2021; Cooper et al., 2016).

In line with the concept of DMs being the final step through which more distal influences are mediated (Cooper et al., 2016), the present study found that mostly-heterosexual men’s indicators of alcohol use were fully or almost fully mediated through their DMs. About half of their AUD symptoms were mediated through DMs. When considering DMs’ specific contributions, the pathways with the largest effect sizes were through enhancement DMs, whereas the pathways through coping DMs were considerably smaller; a small but significant protective pathway was found through conformity DMs to all aspects of alcohol use. This finding indicates that mostly-heterosexual men reported higher levels of conformity DMs, which were negatively associated with indicators of alcohol use. This protective effect of conformity DMs aligns with previous literature (for an overview, see Bresin and Mekawi, 2021; Cooper et al., 2016). For mostly-heterosexual men, elevated conformity DMs may represent an adaptive strategy to mitigate social judgment by aligning with perceived norms, thereby reducing the risk of overt stigmatization. While this can be understood as a coping mechanism that lowers risky alcohol use through adherence to social expectations, further research is necessary to elucidate the nuances of this relationship.

The finding that, for mostly-heterosexual men, the strongest pathways to indicators of alcohol use were mediated through enhancement DMs, while pathways through coping DMs were considerably smaller—or, in the case of AUD, similar for both enhancement and coping DMs—may appear contradictory to the literature, which suggests that the relationships between discrimination based on sexual orientation or general minority status, bi-negativity, stigma-related stress, internalized stigma, and alcohol use are primarily mediated through coping DMs (Feinstein and Newcomb, 2016; Hatzenbuehler et al., 2011; Lewis et al., 2016; Lewis et al., 2017; Schulz et al., 2022b; Wray et al., 2016). However, most of these studies focused solely on coping DMs (i.e., internal negative reinforcement) and did not test for other pathways mediated through external and/or positive reinforcement, such as enhancement DMs. Due to the positive zero-order correlations between the four broad DMs as conceptualized by Cooper (1994), considering only one of them may result in misleading conclusions (Bresin and Mekawi, 2021; Cooper et al., 2016). The presence of enhancement DMs in mostly-heterosexual men is not necessarily in opposition to the explanation of alcohol use levels in sexual minority populations being due to minority stress and bi-negativity (Bostwick and Hequembourg, 2014; Meyer, 2003, 2007). Different individuals may react to negative emotions either by using alcohol as a coping mechanism (coping DMs) or by actively seeking positive emotions (enhancement DMs) as a way to compensate (Waugh, 2020; Waugh et al., 2020).

Thus, to fully understand the aetiological pathways to alcohol use and AUD symptoms and to define treatment targets it is necessary to widen the focus and include other DMs. Interventions tailored to DMs and personality factors have shown promising results (Canale et al., 2015; Conrod et al., 2013; Lammers et al., 2017; Newton et al., 2016; Wurdak et al., 2016). Based on a comprehensive meta-analysis (Bresin and Mekawi, 2021), it was noted that enhancement DMs were strongly linked to alcohol-related problems through increased alcohol use. On the other hand, the link between coping DMs and alcohol-related problems is mainly due to a more generalized tendency of maladaptive avoidance and less due to increased alcohol use.

Consequently, interventions against enhancement DMs should focus on providing alternative sources of positive reinforcement and stimulation, as well as on psychoeducation regarding alcohol’s enhancing effects (Bresin and Mekawi, 2021; Cooper et al., 1995; Correia et al., 2011; Urbán et al., 2008). However, interventions to improve coping DMs may be more effective when addressing general tendencies of maladaptive avoidance, such as through life skills training, improving self-esteem or regulating negative affect (Botvin, 2000; Bresin and Mekawi, 2021; Cooper et al., 1995; Kuntsche et al., 2010; Németh et al., 2011; White et al., 2016; Zaso and Read, 2020).

Contrary to more general studies that have investigated binary differences between sexual minority and heterosexual individuals (e.g., King et al., 2008), the present study did not find higher levels of alcohol use or AUD symptoms among bisexual, mostly-homosexual or homosexual men than among heterosexual men. Indeed, homosexual men even scored lower than heterosexual men on some alcohol use indicators (quantity, frequency, HED). Considering the small effect sizes, this finding seems not only to be due to the small sample but also to indicate that the variations were not only statistically insignificant but also practically negligible. Moreover, our findings do not necessarily conflict with earlier findings in that bisexual, mostly-homosexual and homosexual men are affected by minority stress and bi-negativity. Two recent studies, also using the present research sample, found significantly poorer mental health scores (e.g., depression, stress, satisfaction with life) for this combined population (Marmet et al., 2021; Wicki et al., 2021). While members of the sexual minority population in the United States often rely on heavy-drinking venues as their primary locations for socializing (Bloomfield et al., 2011; Coulter et al., 2016; Parks and Heller, 2013; Trocki et al., 2009), it seems that sexual minority men in Switzerland may now use the internet instead of bars and heavy-drinking venues as their primary ‘location’ for socializing (Wicki et al., 2021). This suggests that cultural context plays a crucial role in influencing sexual minority men’s patterns of socialization and associated behaviors, underlining the necessity to consider cultural variations when examining substance use within these communities. Nonetheless, in Switzerland, alcohol use in the general population follows similar patterns to those observed in other European countries (Suter et al., 2023). Notably, the prevalence of HED is highest in late adolescence and early adulthood compared to other age groups; HED is often intentional, driven by the desire to seek excitement, have fun, and feel the effects of alcohol, and it predominantly occurs in social settings such as bars, pubs, discos, or at special events like festivals (Kuntsche and Gmel, 2013).

Compared to heterosexual men, homosexual men reported consuming smaller usual quantities of alcohol and less frequent alcohol consumption and HED. However, these associations were not mediated through DMs, indicating that overall, homosexual men were not less motivated to consume alcohol but that factors beyond DMs explained this association. A similar pattern has been found in a large cross-cultural study, where gender differences in alcohol use were not mediated through DMs (Kuntsche et al., 2015). Other variables, therefore, such as differences in health consciousness and social and environmental factors (e.g., perceived drinking norms), are plausible explanations, although this needs closer examination. Nonetheless, when looking at specific DMs, greater endorsement of coping DMs was found among homosexual men than among heterosexual men, which might be an indicator of minority stress (Meyer, 2003, 2007).

The present study had some limitations. First, due to its cross-sectional design, no conclusions about causal relationships can be drawn. Second, the questionnaire item on sexual orientation only considered the aspect of sexual attraction (Patterson et al., 2017), and the response options were only suitable for allosexual (i.e., non-asexual) participants, as the spectrum ranged from heterosexual to homosexual. Asexual participants probably skipped the question or selected a less-than-optimal response. Furthermore, all the study participants were summoned to military recruitment based on their sex in official records (which are binary in Switzerland), which may have resulted in misclassifying a small percentage (probably <1%) of transgender women as men (Arcelus et al., 2015; Flores et al., 2016), leading to an incorrect labeling of their sexual orientation. Third, the sample was only representative of Swiss men in a specific age cohort and from French-speaking and German-Speaking regions, so generalizations to women, other age groups, other linguistic regions or other nationalities living in Switzerland should be done with caution. However, sexual minority women have often been reported to be at similar or higher risks of problematic substance use to sexual minority men (Plöderl and Tremblay, 2015; Ross et al., 2018). The present study did not include non-Swiss men residing in the country and, as many of these originate from countries with higher societal levels of homophobia than Switzerland (Lamontagne et al., 2018), it could be assumed that they experience similar or higher levels of minority stress and bi-negativity (Bostwick and Hequembourg, 2014; Meyer, 2003, 2007). However, unlike previous studies exploring the role of DMs to understand alcohol use in the context of sexual orientation, the present study used a non-self-selective, general population sample and thereby aimed to avoid potential biases (Salway et al., 2019). Finally, the present study did not adjust for variables other than age or linguistic region; future research should investigate the interplay of factors such as socioeconomic status and mental health indicators with DMs and alcohol use in the context of sexual orientation.

In conclusion, the present study highlighted the considerable variability in alcohol use and AUD symptoms across the spectrum of sexual orientations and within the population of young sexual minority Swiss men. The small effect sizes for direct and indirect pathways reported indicate that the differences in alcohol use and AUD symptoms across that spectrum were considerably smaller than the differences found in earlier studies based mostly on North American samples (e.g., King et al., 2008; Plöderl and Tremblay, 2015). Nonetheless, our study suggests that higher alcohol consumption among mostly-heterosexual men than among heterosexual men is largely mediated through DMs, particularly through enhancement DMs rather than through coping DMs (as has commonly been assumed), which only mediated alcohol use to a small extent. To gain a better understanding of the aetiological pathways and important differences in subgroups of sexual minorities, it will be crucial for future research to (a) avoid combining subgroups when analyzing sexual orientation (particularly in systematic reviews and meta-analyses) and (b) expand its focus beyond internal, negative reinforcement DMs for alcohol use by considering a broader range of DMs that involve external and/or positive reinforcement (i.e., including DMs other than coping DMs exclusively). From a practical standpoint, this study underscored the importance of addressing sexual orientation in primary care situations and, more specifically, in psychiatric care (Nuñez and Jäger, 2011). Understanding alcohol use differences across the spectrum of sexual orientations, their related risks and their associated aetiological pathways will enable clinicians to tailor more effective and efficient prevention efforts.

## Data Availability

The datasets presented in this study can be found in online repositories. The names of the repository/repositories and accession number(s) can be found at: Doi:10.5281/zenodo.5469953.

## Footnotes

^1^ www.c-surf.ch
