# Supplementary material for: How drinking motives mediate associations between sexual orientation and indicators of alcohol use – a study among young Swiss men
Source: Front Psychol. 2025 Jan 20;15:1416062. doi: 10.3389/fpsyg.2024.1416062 (PMC11788414; doi:10.3389/fpsyg.2024.1416062)
Supplement: Supplementary file 1 [file Table_1.docx]

Supplementary Material

**Supplementary Table 1.** Model fit indices.

|  | **Root mean square error  of approximation  (RMSEA, 95% CI)** | | | **Comparative fit index (CFI)** | **Tucker–Lewis index (TLI)** |
| --- | --- | --- | --- | --- | --- |
| Quantity | **.046** | **[.043;** | **.048]** | **.964** | *.949* |
| Frequency | **.046** | **[.044;** | **.049]** | **.964** | *.949* |
| Volume | **.047** | **[.044;** | **.049]** | **.963** | *.948* |
| HED | **.036** | **[.033;** | **.038]** | *.942* | *.911* |
| AUDIT-C | **.036** | **[.033;** | **.038]** | *.940* | *.910* |
| Maximum | **.046** | **[.044;** | **.048]** | **.964** | *.950* |
| AUDS | **.041** | **[.040;** | **.043]** | *.929* | *.917* |

Note: HED = heavy episodic drinking; AUDIT-C = Alcohol Use Disorders Identification Test-Consumption score; AUDS = alcohol use disorder symptoms.
Bold = good fit; italics = acceptable fit.
