# Supplementary material for: How drinking motives mediate associations between sexual orientation and indicators of alcohol use – a study among young Swiss men
Source: Front Psychol. 2025 Jan 20;15:1416062. doi: 10.3389/fpsyg.2024.1416062 (PMC11788414; doi:10.3389/fpsyg.2024.1416062)
Supplement: Supplementary file 2 [file Table_2.docx]

Supplementary Material

**Supplementary Table 2.** Descriptive statistics of variables across the spectrum of sexual orientation.

|  | **all** | | | **heterosexual** | | | **mostly-heterosexual** | | | **bisexual** | | | **mostly-homosexual** | | | **homosexual** | | |
| --- | --- | --- | --- | --- | --- | --- | --- | --- | --- | --- | --- | --- | --- | --- | --- | --- | --- | --- |
|  | n | m (SD) / % | | n | m (SD) / % | | n | m (SD) / % | | n | m (SD) / % | | n | m(SD) / % | | n | m (SD) / % | |
| **Drinking motives** |  |  |  |  |  |  |  |  |  |  |  |  |  |  |  |  |  |  |
| Age | 5139 | 25.45 | (1.24) | 4521 | 25.44 | (1.24) | 341 | 25.38 | (1.24) | 53 | 25.31 | (1.11) | 29 | 25.00 | (0.81) | 117 | 25.43 | (1.22) |
| Linguistic region | 5139 | 42.5% | | 4521 | 42.7% | | 341 | 46.3% | | 53 | 54.7% | | 29 | 34.5% | | 117 | 42.7% | |
|  |  |  |  |  |  |  |  |  |  |  |  |  |  |  |  |  |  |  |
| **Drinking motives** |  |  |  |  |  |  |  |  |  |  |  |  |  |  |  |  |  |  |
| Social | 5060 | 2.85 | (1.04) | 4513 | 2.85 | (1.04) | 341 | 2.91 | (1.01) | 52 | 2.53 | (1.07) | 29 | 2.97 | (0.99) | 117 | 2.75 | (0.97) |
| Enhancement | 5062 | 2.76 | (1.03) | 4515 | 2.74 | (1.03) | 340 | 2.98 | (1.06) | 53 | 2.62 | (1.12) | 29 | 2.75 | (0.87) | 117 | 2.71 | (1.03) |
| Coping | 5060 | 1.69 | (0.81) | 4512 | 1.67 | (0.80) | 341 | 1.85 | (0.85) | 53 | 1.86 | (0.92) | 29 | 1.70 | (0.62) | 117 | 1.88 | (0.84) |
| Conformity | 5061 | 1.35 | (0.63) | 4513 | 1.34 | (0.62) | 341 | 1.49 | (0.77) | 53 | 1.37 | (0.69) | 29 | 1.46 | (0.64) | 117 | 1.43 | (0.68) |
|  |  |  |  |  |  |  |  |  |  |  |  |  |  |  |  |  |  |  |
| **Alcohol use** |  |  |  |  |  |  |  |  |  |  |  |  |  |  |  |  |  |  |
| Quantity | 5138 | 3.76 | (2.36) | 4520 | 3.76 | (2.37) | 341 | 3.99 | (2.48) | 53 | 3.73 | (2.14) | 29 | 3.76 | (1.85) | 117 | 3.06 | (1.84) |
| Frequency | 5138 | 94.01 | (76.97) | 4520 | 92.64 | (76.09) | 341 | 105.20 | (78.96) | 53 | 112.23 | (105.11) | 29 | 99.52 | (92.31) | 117 | 92.38 | (77.14) |
| Volume | 5138 | 7.73 | (9.40) | 4520 | 7.61 | (9.29) | 341 | 9.07 | (9.77) | 53 | 10.37 | (16.22) | 29 | 8.38 | (9.30) | 117 | 6.10 | (6.68) |
| HED | 5135 | 17.96 | (41.89) | 4517 | 17.83 | (41.71) | 341 | 18.96 | (37.26) | 53 | 32.62 | (83.74) | 29 | 14.76 | (17.79) | 117 | 10.87 | (14.50) |
| AUDIT-C | 5138 | 5.09 | (2.41) | 4520 | 5.07 | (2.41) | 341 | 5.49 | (2.42) | 53 | 5.21 | (2.55) | 29 | 5.10 | (2.45) | 117 | 4.52 | (2.18) |
| AUDIT-C 4+ | 5138 | 73.1% | | 4520 | 72.8% | | 341 | 77.1% | | 53 | 77.4% | | 29 | 72.4% | | 117 | 64.1% | |
| AUDIT-C 6+ | 5138 | 41.6% | | 4520 | 40.8% | | 341 | 51.9% | | 53 | 39.6% | | 29 | 48.3% | | 117 | 31.6% | |
| AUDIT-C 8+ | 5138 | 17.5% | | 4520 | 17.3% | | 341 | 21.1% | | 53 | 18.9% | | 29 | 17.2% | | 117 | 12.0% | |
| Maximum no. | 5126 | 10.39 | (7.40) | 4509 | 10.36 | (7.34) | 341 | 11.27 | (8.39) | 53 | 9.64 | (7.44) | 29 | 9.76 | (6.57) | 116 | 8.41 | (5.35) |
| AUDS | 5130 | 1.33 | (1.63) | 4512 | 1.28 | (1.59) | 341 | 1.80 | (1.90) | 53 | 1.64 | (2.30) | 29 | 1.59 | (1.64) | 117 | 1.22 | (1.70) |
| AUD mild | 5130 | 34.1% | | 4512 | 32.7% | | 341 | 47.8% | | 53 | 39.6% | | 29 | 48.3% | | 117 | 35.9% | |
| AUD moderate | 5130 | 9.5% | | 4512 | 9.0% | | 341 | 15.8% | | 53 | 13.2% | | 29 | 13.8% | | 117 | 6.8% | |
| AUD severe | 5130 | 2.4% | | 4512 | 2.2% | | 341 | 5.0% | | 53 | 3.8% | | 29 | 3.4% | | 117 | 2.6% | |

Remarks: Linguistic region = linguistic region (Francophone vs Germanophone), HED = heavy episodic drinking; AUDIT-C = Alcohol Use Disorders Identification Test-Consumption score; AUDIT-C 4+/6+/8+ = screened positive for scores ≥4, ≥6, or ≥8 points on the AUDIT-C; AUDS = alcohol use disorder symptoms; AUD mild/moderate/severe = screened positive for mild (≥2 symptoms), moderate (≥4 symptoms), or severe (≥6 symptoms) AUD.
For descriptive statistics, the m (SD) are based on the values before log-transformation; drinking motives and AUDS are based on the mean scores of the respective items.
